# NRF2 Activation Restores Disease Related Metabolic Deficiencies in Olfactory Neurosphere-Derived Cells from Patients with Sporadic Parkinson's Disease

**DOI:** 10.1371/journal.pone.0021907

**Published:** 2011-07-01

**Authors:** Anthony L. Cook, Alejandra M. Vitale, Sugandha Ravishankar, Nicholas Matigian, Greg T. Sutherland, Jiangou Shan, Ratneswary Sutharsan, Chris Perry, Peter A. Silburn, George D. Mellick, Murray L. Whitelaw, Christine A. Wells, Alan Mackay-Sim, Stephen A. Wood

**Affiliations:** 1 National Centre for Adult Stem Cell Research, Eskitis Institute for Cell and Molecular Therapies, Griffith University, Brisbane, Queensland, Australia; 2 Department of Otolaryngology Head and Neck Surgery, Princess Alexandra Hospital, Brisbane, Queensland, Australia; 3 School of Medicine, The University of Queensland, Brisbane, Queensland, Australia; 4 School of Molecular and Biomedical Science (Biochemistry), University of Adelaide, Adelaide, South Australia, Australia; National Institutes of Health, United States of America

## Abstract

**Background:**

Without appropriate cellular models the etiology of idiopathic Parkinson's disease remains unknown. We recently reported a novel patient-derived cellular model generated from biopsies of the olfactory mucosa (termed olfactory neurosphere-derived (hONS) cells) which express functional and genetic differences in a disease-specific manner. Transcriptomic analysis of Patient and Control hONS cells identified the NRF2 transcription factor signalling pathway as the most differentially expressed in Parkinson's disease.

**Results:**

We tested the robustness of our initial findings by including additional cell lines and confirmed that hONS cells from Patients had 20% reductions in reduced glutathione levels and MTS [3-(4,5-dimethylthiazol-2-yl)-5-(3-carboxymethoxyphenyl)-2-(4-sulfophenyl)-2H-tetrazolium, inner salt] metabolism compared to cultures from healthy Control donors. We also confirmed that Patient hONS cells are in a state of oxidative stress due to higher production of H_2_O_2_ than Control cultures. siRNA-mediated ablation of NRF2 in Control donor cells decreased both total glutathione content and MTS metabolism to levels detected in cells from Parkinson's Disease patients. Conversely, and more importantly, we showed that activation of the NRF2 pathway in Parkinson's disease hONS cultures restored glutathione levels and MTS metabolism to Control levels. Paradoxically, transcriptomic analysis after NRF2 pathway activation revealed an increased number of differentially expressed mRNAs within the NRF2 pathway in L-SUL treated Patient-derived hONS cells compared to L-SUL treated Controls, even though their metabolism was restored to normal. We also identified differential expression of the PI3K/AKT signalling pathway, but only post-treatment.

**Conclusions:**

Our results confirmed NRF2 as a potential therapeutic target for Parkinson's disease and provided the first demonstration that NRF2 function was inducible in Patient-derived cells from donors with uniquely varied genetic backgrounds. However, our results also demonstrated that the response of PD patient-derived cells was not co-ordinated in the same way as in Control cells. This may be an important factor when developing new therapeutics.

## Introduction

Parkinson's disease (PD) is a late onset disorder recognisable by a characteristic cluster of motor and non-motor neurological symptoms, attributed to region-specific neurodegeneration, which includes the prominent loss of the dopaminergic nigro-striatal tracts [1]. Idiopathic PD is considered a “complex” disease arising from interactions between environmental risk factors with multiple risk-associated genes in individual patients. Research into PD has utilised several animal models, either based on xenobiotic exposure, genetic manipulation, or a combination of both to induce PD-like phenotypes [reviewed in 2]. Whilst much has been learnt from such models, those available are not without limitations. In particular, the toxin-based models, while reproducing the nigro-striatal deficit, fail to replicate the classic pathological hallmarks and the insidious, progressive nature of the human disease. The genetic models also fail to phenocopy the human disease in that they generally lack a parkinsonian behavioural phenotype and/or the characteristic pathological features of the human disease. Moreover, there is a distinct inability of either approach to model sporadic, late-onset disease, which accounts for over 90% of human cases [3], which highlights the need for alternative, but complementary approaches.

The symptoms of PD include neuronal dysfunction in regions outside the nigro-striatal pathway. For example, defects in olfactory function are at least as common as movement dysregulation in PD [4]. The olfactory mucosa, the organ of smell in the nose, is a neuroepithelium that regenerates throughout life producing neurons and glia, and is accessible by biopsy in human adults [5], [6]. We have used these features to establish a novel cellular model of PD, termed human olfactory neurosphere-derived (hONS) cell lines, from olfactory mucosa biopsies from multiple PD patients and healthy Controls [7].

Molecular mechanisms underlying the phenotypes observed in sporadic PD are as yet unknown, but several have been implicated, including decreased levels of glutathione (GSH), proteasomal impairment, oxidative damage and mitochondrial dysfunction [1], [3], [8]. These are not mutually exclusive and the causes of PD are likely to be multifactorial with pathways acting in concert to cause degeneration. This is supported by the observation that the *PARK* proteins which are altered in familial PD cases, are involved in both mitochondrial and proteasomal regulation [3], [9]. Assessment of hONS cells metabolic function in PD compared to Controls hONS cultures revealed decreases in both MTS metabolism and reduced GSH content [7], results consistent with PD post-mortem brain specimens [10], [11], [12]. MTS metabolism is a generalised measure of cellular metabolic activity, based on reduction by NAD(P)H-dependent dehydrogenase enzymes [13], [14], whereas reduced glutathione levels are a marker of oxidative stress buffering capacity [15]. Importantly, these same metabolic functions were specific for PD, as they were unaltered in hONS cultures derived from schizophrenia patients [7]. Thus, the hONS cellular model captures differences in idiopathic Parkinson's disease, based on comparison of multiple cell lines from PD patients and healthy Controls, thereby identifying disease-specific differences on a background of genotypic and phenotypic variability reflective of that within the normal human population.

Gene expression profiling of PD and Control hONS cells revealed a significant dysregulation of pathways with relevance to Parkinson's disease, with the most highly ranked of these being the ‘NRF2-mediated oxidative stress response’ [7]. NRF2 is transcription factor that induces expression of detoxification enzymes such as the NAD(P)H dehydrogenase NQO1 and glutathione synthetic enzymes in response to oxidants and reactive oxygen species by binding to the antioxidant response element (ARE) within gene promoters [16]. Under basal conditions, NRF2 is retained in the cytoplasm by KEAP1, a protein that co-ordinates ubiquitylation of NRF2, thereby promoting its degradation [17]. The KEAP1 - NRF2 interaction is inhibited under oxidative stress, allowing NRF2 to enter the nucleus and induce antioxidant gene expression, including genes that synthesize glutathione. The protective effects of the NRF2 transcription factor against PD-associated toxins such as MPTP and rotenone has been an area of intense research activity. Multiple studies have been conducted in rodent or transformed (e.g. neuroblastoma) human cell line models culminating in NRF2 being touted as a ‘novel therapeutic target’ for PD intervention [18] due to the ability to pharmacologically inhibit the KEAP1 - NRF2 interaction (for example, by treatment with L-Sulforaphane [L-SUL, reference 19]) and thereby concomitantly inhibit proteasomal degradation of NRF2, and increase NRF2 target gene expression. Notably, neuronal cultures from *Nrf2*-null mice also show an approximate 15% decrease in basal glutathione levels compared to control neurons [20], and depletion of glutathione specifically within catecholaminergic neurons results in an age-related neurodegeneration in mice [21]. Post-mortem brain studies are also consistent with a role for NRF2 in PD, as it is detected in the nucleus of remaining neurons within the substantia nigra [22], suggesting that the remaining cells attempt to induce downstream detoxification mechanisms.

Despite these studies, the extent and nature of cellular responses to NRF2 activation have not been established for any PD patient-derived model. Here, we focused on the functional rescue of the GSH and MTS metabolism deficiencies detected in hONS cells from PD patients, by starting with interrogation of the NRF2 pathway, previously identified as dys-regulated in this model [7]. We show for the first time that Parkinson's disease patient cells responded to NRF2 activation, such that cellular metabolic function was restored to levels seen in the Control donor-derived cells. Critically, through assessment of cell lines established from multiple unrelated idiopathic Parkinson's disease patients, we also determined that the spectrum of transcriptional responses to NRF2 activation differed in PD hONS cultures compared to Controls.

## Results

### PD patient-derived hONS cells showed deficiencies in Glutathione and MTS metabolism

Previously we reported decreased levels of reduced glutathione and MTS metabolism in hONS cultures derived from PD patients when compared to Control cultures [7]. To test the robustness of these initial results, here we extended this analysis to a total of 54 donors: 26 Control and 28 PD. Although there was overlap between the two groups, there was a 16% decrease in reduced glutathione (p = 0.016) and 18% decrease in MTS metabolism (p = 0.019) in PD-derived hONS cells (Fig S1). Other cell metabolic functions (ATP content, chymotrypsin-like proteasome activity, membrane integrity (LDH activity) and caspase-3/7 activation) did not differ between Control- and PD-derived cultures (Fig S1). Potential confounders of these group differences were either controlled for, or tested post-hoc (Fig S2). There were similar numbers of male (Control N = 15; PD N = 17) and female (Control N = 11; PD N = 11) donors in each group, average age was not significantly different between groups, and there was no correlation of any metabolic function with donor age or cell line passage number.

These results raised the possibility that PD Patient cells are in a state of oxidative stress. To quantify this directly, we then measured the production of H_2_O_2_ in Control and PD cultures (N = 8 in each group) at several time-points post-seeding (Fig S1G). At 24 hr post-seeding, Control and Patient hONS cultures had similar levels of H_2_O_2_. For both Control and Patient cultures, the amount of H_2_O_2_ in the medium increased over time, however after 48 hrs, H_2_O_2_ levels were statistically significantly higher in PD cultures, and this difference remained significant at all other time-points (72 and 96 hr). This suggests that PD Patient hONS are in a state of oxidative stress.

### NRF2 silencing recapitulates PD-associated metabolic deficiencies in Control hONS cells

**Figure 1:**
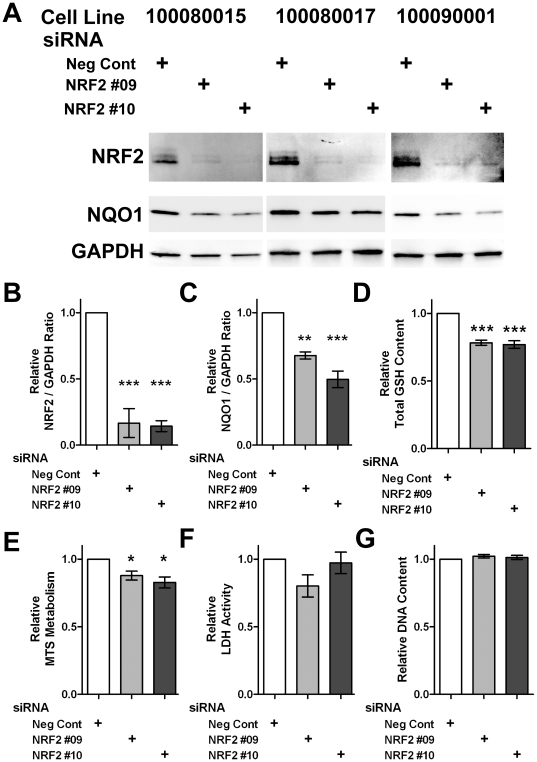
NRF2 depletion recapitulates PD-associated changes in metabolic function. **A.** Immunoblot analysis of 3 Control hONS cell lines transiently transfected with either negative control (Neg Cont) siRNAs or siRNAs targeting NRF2 (NRF2 #09 and NRF2#10). The protein being detected is indicated to the left of the gel panels. **B–C**. Quantification of NRF2 and NQO1 protein levels relative to GAPDH protein in cultures described in A. **D–G.** Total GSH content (D) and MTS metabolism (E) were both statistically significantly decreased in Control hONS transfected with NRF2 targeting siRNAs compared to negative control siRNAs. LDH activity (F) and DNA content (G) were not affected by decreased NRF2 function. For B–G, statistical significance was determined using ANOVA with Dunnett's multiple comparison post-test with the following thresholds: *, p<0.05; **, p<0.01, ***, p<0.001.

To determine if decreased NRF2 function could account for the decreased GSH and MTS metabolism observed in PD hONS cultures, we silenced NRF2 using two NRF2-targeting siRNA sequences (NRF2 #09 and NRF2 #10) in three different Control hONS cultures (Fig 1). Decreased NRF2 protein levels were confirmed by immunoblot of total protein lysates, and showed an average 83% and 86% knockdown for NRF2 #09 and NRF2 #10 siRNAs respectively, when compared to negative control transfected cells after normalisation of NRF2 protein levels to GAPDH levels (Fig 1A,B). NQO1, a detoxification enzyme down-stream of NRF2 [16] was also decreased by ablation of NRF2 (32% and 50%; Fig 1A,C). Assessment of metabolic function in cells transfected in parallel revealed that both total glutathione content (22% and 23% decreases, Fig 1D) and MTS metabolism (12 and 17% decreases, Fig 1E) were affected by NRF2 depletion. These decreased metabolic functions were consistent for each NRF2 targeting siRNA used, and were statistically significantly different from negative control siRNA cultures. In contrast, cell membrane integrity (LDH activity, Fig 1F) and total cell number (as determined by DNA content, Fig 1G) were unaffected. These results suggest that decreased NRF2 function may be responsible for the diminished glutathione and MTS metabolism levels observed in PD hONS cultures.

### NRF2 pathway induction in Patient hONS-derived cells alters cell metabolism

**Figure 2:**
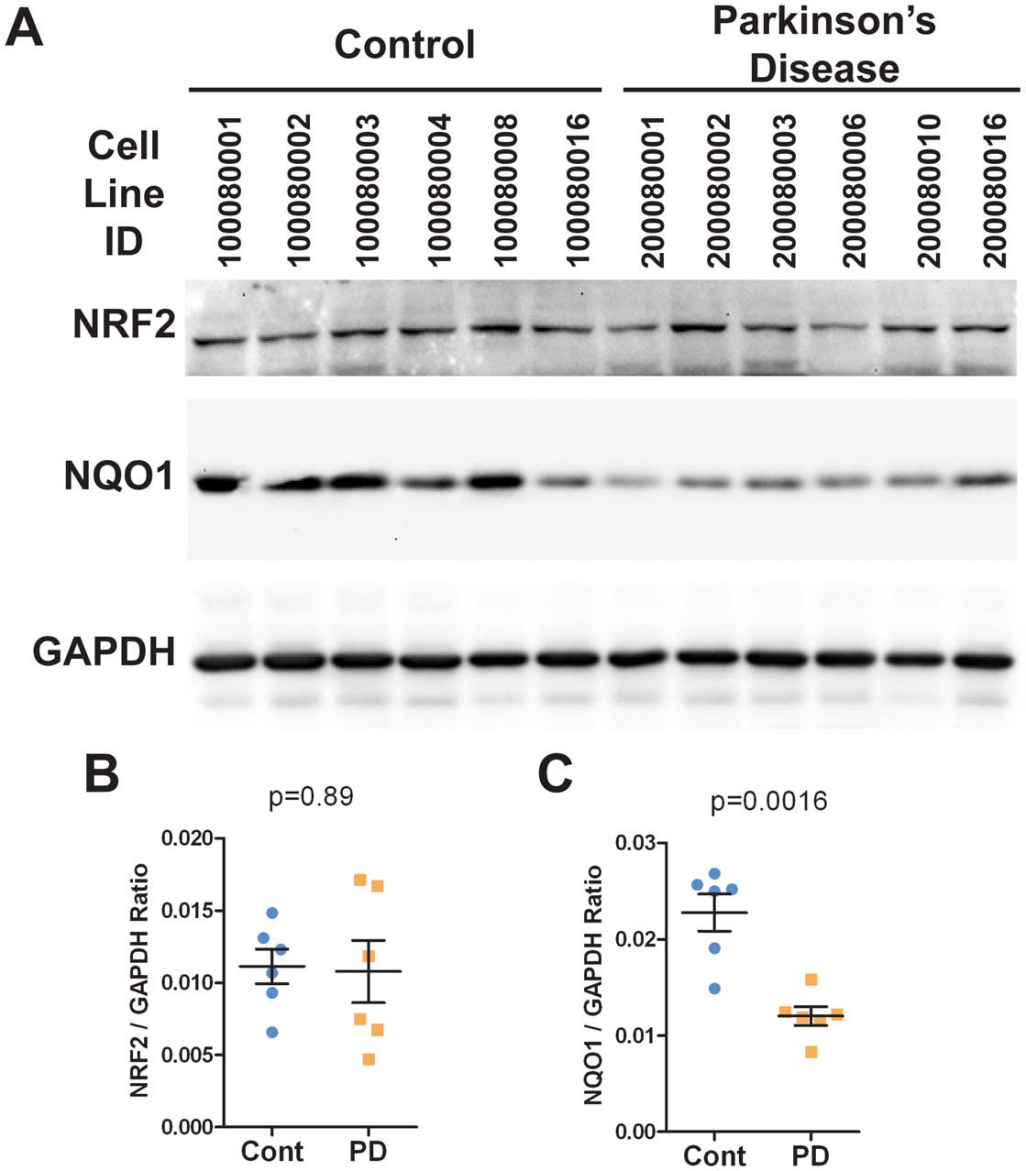
NRF2 pathway components are expressed in hONS cultures. **A.** Immunoblot analysis of total protein lysates from 6 Control donor-derived and 6 PD patient-derived hONS cell lines. The protein being immunodetected is indicated to the left of each gel panel. **B–C.** Quantitation of NRF2 (B) and NQO1 (C) protein levels relative to GAPDH.

Immunoblot analysis of total protein lysates of hONS cultures revealed that expression of the NRF2 transcription factor was similar in both Control and PD cells (Fig 2A,B). However, levels of the NRF2 target NQO1 were decreased by approximately 50% (p = 0.0016) in the PD hONS cell lines examined (Fig 2A,C). This result, combined with the effects of NRF2 ablation in Control hONS cultures (Figure 1), suggested that activation of the NRF2 pathway in PD hONS cultures may be a valid approach to restoration of the disease-associated transcriptional and metabolic deficiencies to Control hONS culture levels.

**Figure 3:**
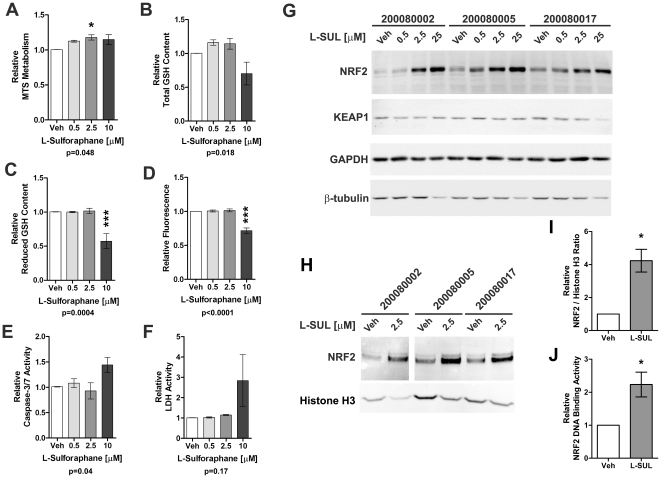
Dosage effects of L-SUL on cell metabolic function and NRF2 in PD-derived hONS cell metabolism. **A–F.** PD patient-derived hONS cultures (N = 4) were treated with the indicated doses of L-SUL for 24 hr and assessed for MTS metabolism (A), total glutathione (GSH) content (B), reduced GSH content (C), DNA content (D), caspase-3/7 activity (E) and membrane integrity (LDH activity, F). Bars show mean and s.e.m., with data presented as fold change compared to vehicle treated cultures for each cell line. For A-F, data was analysed by one-way ANOVA using Dunnett's multiple comparison test. The p value at the bottom of each panel indicates the ANOVA result with p<0.05 considered significant. The asterisks above show the results of Dunnett's multiple comparison post-tests comparing all L-SUL treated cultures to vehicle (Veh) treated cultures; * p<0.05, *** p<0.001. **G.** Total protein lysates from three hONS cell lines were analysed by immunoblot for NRF2 and KEAP1. GAPDH and β-tubulin were used as loading controls. The protein being immunodetected is indicated to the left of each gel, and the L-SUL dose and cell line are indicated above the blot panels. **H**. Nuclear extracts of three hONS cell lines were analysed by immunoblot for NRF2 levels using Histone H3 as a loading control. The protein being immunodetected is indicated to the left of each gel, and the L-SUL dose and cell line are indicated above the blot panels. **I.** Quantitation of NRF2 nuclear accumulation in PD hONS cultures relative to Histone H3 protein levels; p = 0.021 using one-tailed Student's t-test. **J.** NRF2 DNA binding to the ARE motif was quantified in nuclear fractions of DMSO and L-SUL treated PD hONS cell lines; p = 0.041 using one-tailed Student's t-test.

We subsequently examined whether activation of the NRF2 pathway with L-SUL would increase the glutathione content and MTS metabolism in PD hONS cells to Control levels. Treatment of 4 PD hONS cultures for 24 hr with L-SUL (0.5 or 2.5 µM) produced an approximate 12 and 18% increase in MTS metabolism respectively (Fig 3A). These doses of L-SUL also increased total glutathione content by 16 and 14% (Fig 3B). At these concentrations, L-SUL treatment was not overtly toxic because there was no difference in caspase-3/7 activity, LDH activity or DNA content compared to when treated with DMSO alone (Fig 3D–F). At 10 µM, L-SUL treatment did have a slight toxic effect, because DNA content decreased approximately 28%, and caspase-3/7 activity and LDH activity were increased by 43% and 180%, respectively. Although MTS metabolism was still elevated, both total and reduced glutathione content were lower with 10 µM L-SUL treatment (30 and 43% respectively, Fig 3B,C).

We confirmed by immunoblot that L-SUL treatment of PD hONS cultures induced a dose-dependent increase of NRF2 levels in three hONS cell lines derived from different PD Patients (Fig 3G). For all three cell lines, we observed robust NRF2 increases at both 2.5 and 25 µM L-SUL treatment. No change in the levels of the NRF2 negative regulator KEAP1 [17] was observed at any concentration of L-SUL used. Levels of GAPDH were unchanged at all concentrations of L-SUL, whereas β-tubulin levels were decreased at 25 µM. We additionally confirmed that L-SUL treatment caused a nuclear accumulation of NRF2 protein. Immunoblot analysis of nuclear extracts prepared from these same PD hONS cell lines treated with L-SUL (2.5 µM for 6 hours) revealed robust nuclear accumulation of NRF2 in all three 3 cell lines at approximately 4 times the level of DMSO cultures, and which also resulted in increased NRF2 DNA binding to an ARE motif (Fig 3H–J). These results are all consistent with the known action of L-SUL in inhibiting KEAP1-mediated proteosomal degradation of NRF2 [19], and thus these changes in metabolic function are likely due to decreased proteosomal degradation of NRF2.

In order to exclude the possibility that the increases in glutathione content and MTS metabolism induced by L-SUL were non-specific, we used two other known NRF2 activators *tert*-butylhydroquinone (tBHQ) and diethyl maleate (DEM) [19]. Treatment of PD and Control hONS cells (N = 3 per group) with 100 µM tBHQ or 50 µM DEM for 24 hr both significantly increased glutathione levels (Fig S3). Similarly, both compounds increased MTS metabolism but the increase was statistically significant only in the PD hONS cells. Regardless of disease state, the level of induction over vehicle treated cultures was similar for each parameter such that tBHQ and DEM produced 60% and 70% increases, respectively, in reduced glutathione levels in Control hONS cells compared to 90% and 80% increases in PD-derived cultures (Fig S3A–B). Similar results were observed for the induction of total glutathione and MTS metabolism levels (Fig S3C–F). Thus, we consistently observed increased levels of MTS metabolism and total glutathione in hONS cell from PD patients through activation of NRF2.

### L-SUL restored metabolic deficiencies in patient-derived hONS cells

**Figure 4:**
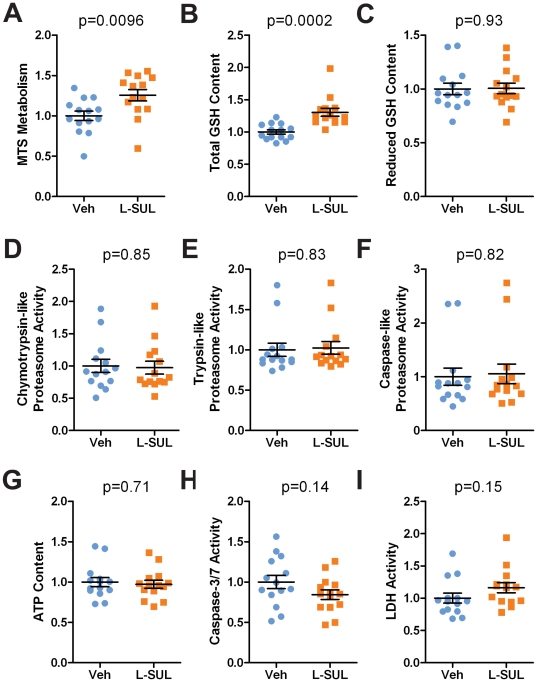
Induction of Glutathione and MTS metabolism in PD-derived hONS cell cultures. **A–I.** PD patient-derived hONS cultures (N = 14) were treated with 2.5 µM L-SUL for 24 hr prior to assessment of cell metabolic functions, including (A) MTS metabolism, (B–C) total and reduced glutathione (GSH) content, (D–F) chymotrypsin-like, trypsin-like and caspase-like proteasome activities, (G) ATP content, (H) caspase3/7 activity and (I) membrane integrity/LDH activity. Each circle (veh) or square (L-SUL treatment) represents data obtained from triplicate plates for an individual cell line. Error bars show mean and s.e.m.. Statistical significance was determined by two-tailed t-test with Welch's correction of vehicle versus L-SUL treated values, with p<0.05 considered significant. Resulting p-values are shown above each graph.

To determine the universality of L-SUL treatment in PD hONS cells we assessed the metabolic responses of 14 PD cultures to 24 hr treatment with 2.5 µM L-SUL. Again we observed that total glutathione and MTS metabolism were increased (Fig 4A–B), whereas no other assessed metabolic function (reduced GSH, proteasome activities, ATP content, caspase-3/7 activity or LDH activity) was altered (Fig 4C–I). Following L-SUL treatment, 86% of the PD patient-derived cell lines (12 of 14) showed an appreciable (at least 10%) increase in MTS and total glutathione levels, relative to vehicle (see Table S1 for individual cell line responses). Of these, 10 cell lines (71%) showed greater than 20% increase in both assays. One cell line (200060004) increased both MTS metabolism and total glutathione content, but only slightly (8% and 7%, respectively), and one cell line (200080013) showed no change. Notably, for the majority of PD hONS cell lines examined, the percentage increase was sufficient to restore the metabolic functions to that of untreated healthy Control hONS cultures (Fig S1 and [7]). Interestingly, the observed increases in total glutathione were not accompanied by increased levels of reduced glutathione which remained constant, or even slightly decreased in some lines (Fig 4C).

Metabolic changes produced by L-SUL treatment of Control hONS cell lines were similar to those in PD hONS cell lines. In 17 Control cultures treated with 2.5 µM L-SUL for 24 hr, there was a 25% (range 9–45%) increase in total glutathione and 30% increase (range 15–48%) increase in MTS metabolism (Fig S4A–B) compared to DMSO treated cultures. As with PD-derived hONS cultures, all other metabolic functions remained unchanged (Fig S4C–I). Immunoblot analysis of 2 Control and 3 PD hONS cultures treated with L-SUL showed similar levels of NRF2 accumulation compared to DMSO treated cultures (Fig S4J). For all cultures examined, this also resulted in increased NQO1 protein levels (Fig S4J). Thus, using three different NRF2 stabilising compounds (L-SUL, tBHQ and DEM), we observed that Control and PD hONS cultures responded in a similar manner in terms of both magnitude of response and the spectrum of metabolic functions that responded.

### NRF2 pathway activation increases differential gene expression in PD and Control hONS cells

**Figure 5:**
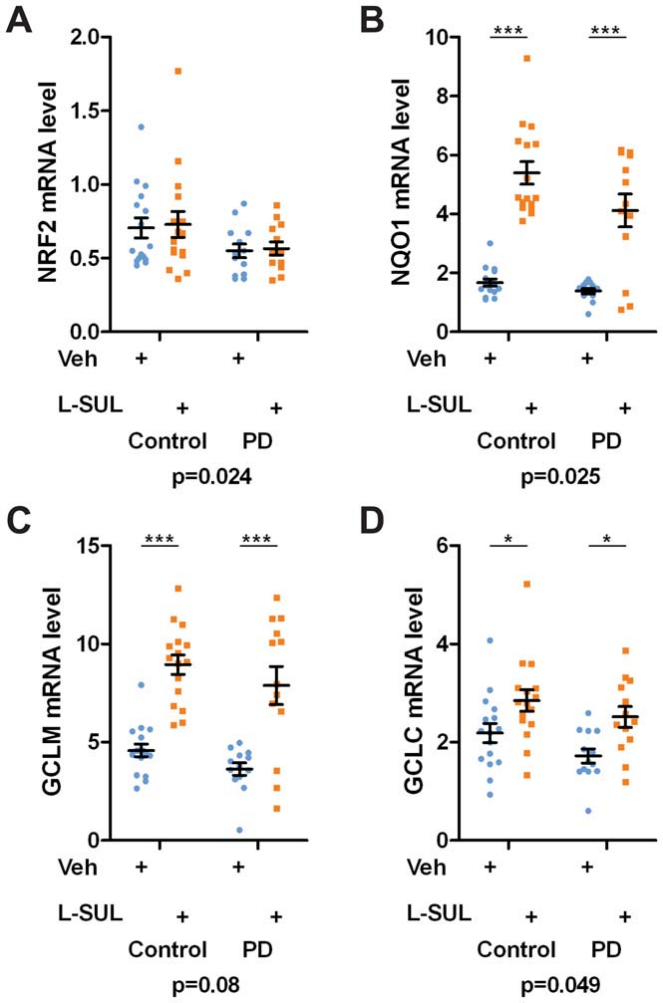
Basal and L-SUL induced levels of NRF2 targets genes in hONS cells. **A–D.** hONS cultures established from Control donors (N = 16) or from PD patients (N = 13) were treated either with vehicle (circles) or 2.5 µM L-SUL (squarers) for 24 hr prior to RNA extraction. The mRNA levels of *NRF2* (A), or the NRF2 target genes *NQO1*, *GCLC* and *GCLM* (B–D) were measured by real-time PCR of cDNA and are presented relative to *EEF1A1*. Statistical significance was determined by 2-way ANOVA. The p value at the bottom of each panel indicates the ANOVA results comparing Control to PD hONS cultures; p<0.05 was considered significant. The asterisks above show the results of Bonferroni post-tests comparing DMSO and L-SUL treated cultures for either Control or PD hONS cell; * p<0.05, *** p<0.001.

To determine the extent of changes in NRF2 target gene expression, RNA samples were prepared from 13 PD and 16 Control hONS cultures treated for 24 hr with 2.5 µM L-SUL or DMSO and assessed by qRT-PCR. In both Control and PD hONS cultures, we observed that L-SUL treatment increased mRNA levels of NRF2 target genes *NQO1*, *GCLC* and *GCLM*, but that *NRF2* gene expression was not altered (Fig 5A–D). All but three PD derived cultures showed greater than 2-fold induction of *NQO1* mRNA levels on L-SUL treatment (see Table S1 for individual cell line responses). The magnitude of *GCLC* and *GCLM* induction was variable between cell lines (Table S1).

We next used whole-genome microarray profiling to examine in more detail the effects of L-SUL treatment on PD and Control hONS cell lines (ArrayExpress Accession Number E-TABM-879). Of the 48,803 probes on the array, 14,836 passed quality control and detection criteria. Using ANOVA on these probes, at a p-value cut off of 0.05, 5% of probes would be significantly different by chance (n = 741). We detected 645 probe differences between vehicle-treated PD and vehicle-treated Control hONS. Significantly however, these were not randomly distributed across different expression pathways but, as previously reported [7], the NRF2 pathway was significantly different between the groups. So far we have analysed a total of 30 PD and 24 Control hONS cell lines in three separate experiments (7, this report and unpublished) and in each the NRF2 pathway is significantly different in PD hONS cells. All other comparisons (see below) were at least three times greater than the 741 predicted by chance. Principal component analysis (Fig S5) of the data demonstrated that L-SUL treatment was the major driver of variance between the four experimental groups.

We hypothesised that L-SUL treatment would decrease the dysregulation of the NRF2 pathway at the mRNA level because it restored metabolic deficiencies in PD hONS cells. mRNA expression data were subjected to ANOVA to assess the impact of L-SUL treatment with main effects being treatment (+ L-SUL) and disease status (PD versus Control). L-SUL treatment induced significant changes in expression of 2320 mRNAs in PD hONS cells compared to 3689 (approximately 1.6 fold more) in Control cells (Table S2). Of these, 1075 were independent of disease state and all but 16 had the same direction of change. In vehicle treated cultures, 645 probes (Table S3) were differentially expressed between PD and Control cultures, of which 219 were also differentially expressed in the L-SUL treated cells. However, in L-SUL treated cultures approximately four times as many mRNAs displayed a significant difference between PD and Control cultures (N = 2647, see Table S4).

**Figure 6:**
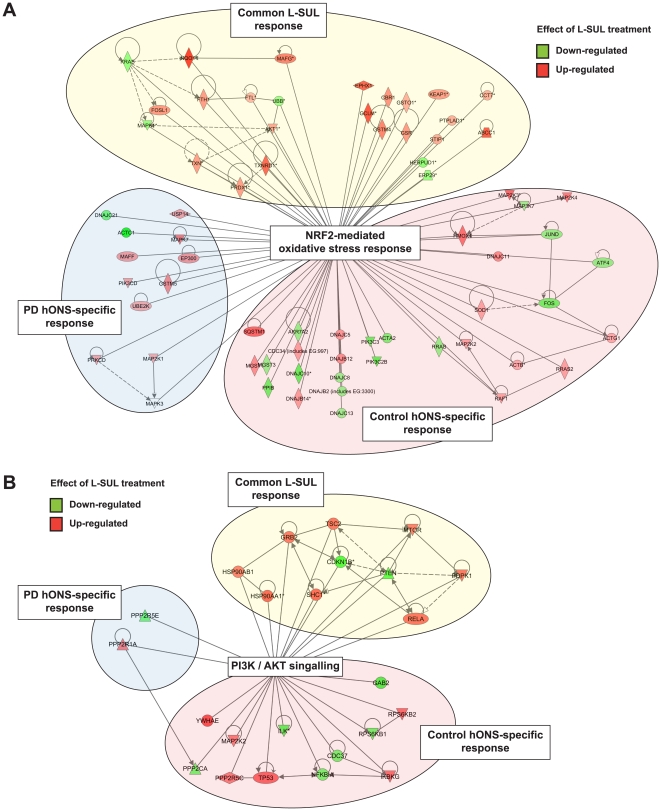
Transcriptomic analysis identified discrete mRNA sets induced by L-SUL in Control versus PD hONS cultures. **A.** Graphical representation of the ‘NRF2-mediated oxidative stress response’ Ingenuity pathway annotation mRNA set indicating mRNAs either induced (red icons) or repressed (green icons) by L-SUL treatment. Three discrete clusters of mRNA expression level changes were able to be identified: i) PD-specific (blue shading), ii) Control hONS-specific (red shading) and iii) those common to both PD and Control hONS cultures (yellow shading). **B.** Graphical representation of the ‘PI3K / AKT signalling’ Ingenuity pathway annotation mRNA set indicating mRNAs either induced (red icons) or repressed (green icons) by L-SUL treatment. Three discrete clusters of mRNA expression level changes were able to be identified: i) PD-specific (blue shading), ii) Control hONS-specific (red shading) and iii) those common to both PD and Control hONS cultures (yellow shading).

Geneset enrichment analysis using Ingenuity pathway annotations revealed that the ‘NRF2-mediated oxidative stress response’ was the predominant pathway that was differentially expressed between Control and PD cultures treated with DMSO (p = 7.76E-5) thereby replicating our initial finding [7]. Strikingly, the ‘NRF2-mediated oxidative stress response’ was also the most dysregulated pathway between Control and PD cultures after L-SUL treatment (p = 2.14E-9; Table S5), with many more mRNAs in this pathway differentially expressed after treatment (47 mRNAs) than between cultures treated with DMSO (15 mRNAs; see Fig S6A). Consistent with genome-wide differences in the number of mRNAs responsive to L-SUL treatment in PD and Control cultures, we again found that approximately 1.5 times as many mRNAs within the ‘NRF2-mediated oxidative stress response’ pathway showed altered expression level in Control cultures after L-SUL treatment compared to Patient cultures. The differentially expressed NRF2 pathway genes could be classified into three groups: (i) those altered in both cell types, (ii) those altered in PD cells alone, and (iii) those altered in Control cells alone (Fig 6A). Many genes in each group are established NRF2 target genes, and included *NQO1* and *GCLM* mRNAs (consistent with our qRT-PCR results) and other known NRF2 targets such as *MAFG* [23], *KEAP1* [24], *GSR* [25], *EPHX1* [26], *TXNRD1* [27], *PRDX1* [28] and *TXN* [29], all of which were induced in response to L-SUL treatment in both PD and Control hONS cultures. Known NRF2 target genes were also altered only in the PD cells (*GSTM5* [30], *USP14* [31]), or only in Control cells (*AKR7A2* [32], [33], *ATF4* [31], *HMOX1* [34], *SQSTM1* [35], *MGST1* and *MGST3* [36]).

Differences in mRNA expression between PD and Control hONS cells after L-SUL treatment were not restricted to down-stream NRF2 target genes, but also included signalling molecules and transcriptional co-regulators that may impact on regulation of NRF2 function. These included transcription factors such as ATF4 [34], JUND [37] and FOS [38], as well as members of the small MAF family [reviewed in 39], all of which can heterodimerise on DNA with NRF2 and influence its transcriptional output. We detected mRNAs for both *MAFG* and *MAFF* in hONS cells, and consistent with previous data reporting NRF2-mediated activation of *MAFG* expression [23], we found that *MAFG* was induced by L-SUL in both Control and PD hONS cultures, whereas *MAFF* was only induced in PD cells (Fig 6A).

L-SUL treatment also induced significant differential expression in other pathways in PD hONS cells compared to Control cells. Among these was the ‘PI3K/AKT signalling pathway’, which was differentially expressed in L-SUL treated cells (p = 1.10E-06), but not in vehicle treated cells (p = 0.37, see Table S5, Figure S6B). Again, mRNAs within this pathway were divisible into three groups: those altered in both cell types, those altered in PD cells alone, and those altered in Control cells alone (Fig 6B). Of note was the relatively large number of mRNAs in this pathway altered in Control cells compared to PD cells. L-SUL treatment did alter some pathways such that they were no longer significantly over-represented in any particular group. For example, pathways involved in amino acid metabolism (‘phenylalanine, tyrosine and tryptophan biosynthesis’ and ‘glycine, serine and threonine metabolism’) were no longer significantly differentially expressed between control and PD-patient hONS cells (Table S5).

Thus, despite restoring identified metabolic deficiencies and some mRNA expression levels in PD hONS cells, L-SUL treatment also reinforced some existing transcriptional differences, and furthermore identified pathways that were only differentially expressed between Control donor and PD-patient derived cell lines post-treatment.

## Discussion

In this study, we show for the first time that activation of NRF2 in a novel patient-derived cellular model of idiopathic Parkinson's disease, previously shown to have a dysregulated NRF2 pathway [7], could rescue disease associated metabolic functional deficits. We firstly replicated findings of decreased levels of reduced GSH and MTS metabolism levels in PD patient cells compared to healthy Control cells in a larger patient cohort, and confirmed a higher state of oxidative stress in Patient cultures. We then showed that deactivation of NRF2 by siRNA in Control donor cells recapitulated the PD-associated changes in metabolic function, but that activation of the NRF2 pathway ‘rescued’ the metabolic phenotype in the PD hONS cells. Importantly, this study is the first to demonstrate a restorative role for NRF2 in regulating disease-associated metabolic deficiencies, including elevation of GSH content, in Parkinson's disease patient-derived cells, providing both validation and a model to further explore NRF2 as a therapeutic target for PD. Interestingly we showed that activation of the NRF2 pathway exacerbated mRNA differences in the same Parkinson's disease patient and Control groups. These disease-specific differences were observed in cells from thirteen unrelated individuals with idiopathic disease compared with sixteen unrelated healthy Control individuals.

In both this and our previous study [7], we have reported differential expression of mRNAs comprising the NRF2-mediated oxidative stress response between Control and PD hONS cell lines under basal culture conditions, and when the NRF2 pathway is activated. These transcriptional differences are supported by altered protein levels and metabolic functions attributable to changes in NRF2 functioning. We have previously proposed that underlying genetic background differences between individual Patients and individual Control cultures are at least in part responsible for these observations [7]. In this regard, the “NRF2-mediated oxidative stress response” is highly polymorphic, with SNPs found within coding regions of multiple detoxification and antioxidant genes regulated by NRF2 [40], within ARE motifs bound by NRF2 within many of these gene promoters [41], and within the *NRF2* protein coding region itself [42]. It may be the different combinations of haplotypes within multiple genes comprising the NRF2 pathway that imparts the cellular phenotype difference between Patient and Control hONS cultures, rather than polymorphism or mutation of a single gene within the pathway. Such a scenario is consistent with the long standing association between polymorphisms within detoxification genes and environmental (e.g. pesticides, solvents) exposures with modifying Parkinson's disease risk [43], [44], [45], [46], [47], and with identification of a protective haplotype within the *NRF2* gene [42].

Whole organism studies have revealed a role for NRF2 in regulating both health and lifespan [reviewed in 48], such that NRF2 function is increased in long-lived species. For example, biochemical analysis of long-lived rodents revealed an increase in total GSH content which was attributed to increased NRF2 protein levels [49]. In contrast, NRF2 function is reduced in the spinal cord astrocytes of aged mice [50] and livers of aged rats [51], and can result in an age-related decrease in GSH, a finding consistent with our results (Fig 1) and analysis of *Nrf2-null* neurons [20], [52]. Similar to our study, NRF2 activity in both of these aged rodent models could be induced by treatment with exogenous chemicals, and resulted in increased GSH content and expression of NQO1 [50], [51]. In the hONS model, NRF2 dysfunction (either by siRNA knockdown, or comparing Patient to Control cultures) reveals a cellular phenotype that is consistent with (i) the diminished function of NRF2 seen in aging, the most established risk factor for Parkinson's disease [53], and (ii) the degenerative nature of the disease. Combined, this suggests that decreased NRF2 activity may be involved in the pathogenesis of Parkinson's disease.

We have shown in this study that activation of NRF2 by L-SUL treatment of PD-patient derived cells restored levels of GSH to Control cell line levels (Fig 3 and 4), and that ablation of NRF2 function in Control hONS cultures decreased GSH content (Fig 1). Decreased levels of GSH are a recurrent finding in Parkinson's disease brain specimens [10], [11], [12], and several clinical trials have evaluated GSH supplementation with the expectation that increased levels would be beneficial to Parkinson's disease patients [54], [55]. Due to its neuroprotective role and ability to increase GSH content, NRF2 activation has been a focal point of research into disease therapies [reviewed in 18,56], although prior to the current study, the effectiveness of this approach in any Patient-derived model had not been examined.

Our data have also revealed a consistent decrease in MTS metabolism in PD hONS cultures (Fig S1 and [7]), an assay considered to reflect cellular activity of NAD(P)H-dependent dehydrogenase enzymes [13], [14]. Cellular reduction of MTS and related compounds is often used to assay cell viability [57], [58], with a decrease in metabolism considered an indication of decreased cell viability. Like GSH content, the decrease in MTS metabolism may also reflect dysfunctional NRF2, as knockdown of NRF2 in Control hONS cultures produced a decrease in MTS metabolism (Fig 1), and which was also induced by L-SUL treatment (Fig 3–4). In other PD models, several dehydrogenase enzymes have been reported to have decreased activity, most notably the mitochondrial Complex I NADH coenzyme-Q dehydrogenase [reviewed in 59]. This defect is not restricted to neurons, and is also observed in other cell types such as platelets [60], [61], [62], and in cell hybrid models of Parkinson's disease [63]. Whilst we have not examined Complex I activity in the hONS models, NQO1, an NAD(P)H dependent dehydrogenase enzyme and direct NRF2 target gene, was shown to be decreased in PD hONS cultures (Fig 2), and this may in part contribute to diminished MTS metabolism in these cells. Decreased MTS metabolism in PD hONS cells, as an assay of cellular dehydrogenase activity [13], [14] and/or viability [57], [58] when coupled with our NRF2 knockdown experiments (Fig 1) is therefore consistent with a degenerative cellular phenotype. Thus, that NRF2 activation by L-SUL, tBHQ or DEM treatment, induced MTS metabolism to Control levels is indicative of restored metabolic function in PD hONS cells.

Our transcriptomic analysis of Control and PD hONS cultures revealed dysregulation of the NRF2-mediated oxidative stress response (Table S5 and [7]). Our data suggests that NRF2 dysregulation in PD hONS cells is not due to alteration of NRF2 protein levels or the ability of NRF2 activity to be induced. Rather, dysfunction may occur in the co-ordination of NRF2-mediated transcriptional activation upon L-SUL-responsive promoters, evidenced by transcriptomic analysis which classified the dysregulated mRNAs into three groups (common-, PD-specific, or Control-specific; see Fig 6A). Given our identification of discrete sets of mRNAs induced by L-SUL in PD and Control hONS cells, including *MAFF* induction by L-SUL being restricted to PD cultures (Fig 6A), it is possible that altered NRF2-small MAF heterodimer formation is driving the differential expression of genes within the NRF2 pathway in hONS cells. Antagonism of NRF2 function by small MAF proteins has been reported to occur in co-transfection experiments using reporter constructs [64]. Consistent with these findings, knockdown of *MAFG* in human hepatocellular carcinoma cells increased NRF2 DNA binding activity and induced GSH synthetic enzymes, resulting in increased GSH content [65]. Moreover, genetic ablation of small *Maf* function in mice has revealed a requirement of these proteins for induction of NRF2-dependent transcription [66]. Thus, the relative abundance of small MAF proteins in a given cell may influence the spectrum of NRF2-dependent transcription, because some target genes appear to have different sensitivities to depletion of specific *Maf* genes [66], [67]. Interestingly, *MafG^−/−^*:*MafK^+/−^* mice display a neuronal degeneration, including aggregations of ubiquitylated proteins, both earlier, and with a higher penetrance than mice null for only *MafG* [67], [68]. Investigation of small MAF function in the hONS model may implicate this family of proteins in Parkinson's disease pathology.

Another novel finding was that L-SUL treatment resulted in significant differential mRNA expression changes within several pathways, in a disease-specific manner. These were not evident in vehicle-treated cultures (Table S5). Whether such pathway responses are a direct consequence of NRF2 dysfunction, or are an NRF2-independent difference in the response of PD hONS cells to L-SUL remains to be determined. Of note however, are some pathways with relevance to Parkinson's disease. Inhibition of PI3K signalling can reduce NRF2 nuclear translocation and antioxidant gene induction in neuroblastoma cells [69], [70], and can also inhibit the antioxidant-induced binding of NRF2 to gene promoters, allowing BACH1 to remain associated with the antioxidant response element and repress transcription [71]. Impaired PI3K/AKT signalling has been reported in *Drosophila* models of PD in which either *DJ-1* or *Parkin* were suppressed by RNAi [72] and in PD patient-derived lymphoblast cultures carrying the DJ-1 L166P mutant [73]. When combined with our current study, these findings suggest convergence of genetic and idiopathic mechanisms of PD manifestation on PI3K signalling.

Whilst our hONS cell model reflects several molecular changes frequently reported in PD [7], it is a possibility that the rescue of deficient metabolic functions in hONS cultures by activation of the NRF2 pathway may not occur in other cell types, specifically neurons. However, the key trigger for the disease may reside in non-neuronal cells. This is evidenced in humans by the development of PD-like changes in previously healthy grafted neurons [74], [75] and in rodent studies that show astrocyte-restricted re-expression of Nrf2 in *Nrf2*-null mice is sufficient to mitigate the neurodegenerative effects of MPTP *in-vivo* [52]. Thus, that we have shown that PD patient derived hONS cells have altered NRF2-mediated responses compared to Controls raises the possibility that NRF2 dys-regulation may be widespread in PD, and not restricted to neurons.

In conclusion, we have for the first time demonstrated that PD patient-derived cells have alterations in NRF2 signalling revealed by cellular responses to activators of this pathway. In our hONS model, NRF2 pathway activation restored disease-specific deficits in cellular functions (glutathione content and MTS metabolism), however gene expression profiling suggests a concomitant further distortion of a disease-altered genetic network. These results further demonstrate the utility of hONS cultures for understanding disease-associated changes in the cell biology of idiopathic Parkinson's disease. In particular, hONS cultures may be suitable for pre-clinical testing of pharmaceuticals targeting NRF2 for disease therapy.

## Materials and Methods

### Ethics Statement

All donor tissue and information was obtained with the informed, written consent of the participants and all procedures were in accordance with National Health and Medical Research Council Code of Practice for Human Experimentation and approved by the Griffith University Human Experimentation Ethics Committee.

### Participants and nasal biopsies

Patients with PD (N = 28) were recruited from consumer groups and through research participant registers maintained by the Queensland Centre for Mental Health Research and the Queensland Parkinson's Project. Controls (N = 26) were recruited from the general population. The patients were diagnosed by a movement disorders neurologist according to UK Brain Bank criteria. Controls completed the same questionnaire as patients to exclude undiagnosed PD. None of the healthy Controls was on PD medication. Olfactory biopsies were obtained by a specialist otorhinolaryngologist according to published protocols [5].

### Cell culture

hONS cell lines from PD patients and healthy Control donors were established as previously described [7]. Olfactory neurospheres were generated from primary cultures of olfactory mucosa plated onto poly-L-lysine (Sigma) coated tissue culture plates in serum-free DMEM/F12 (Invitrogen) medium containing 50 ng/mL EGF (Sigma) and 25 ng/mL FGF2 (Sigma). Neurospheres were collected and dissociated for subsequent propagation as adherent monolayers in DMEM/F12 medium containing 10% FBS (GibcoBRL) to produce hONS cell lines.

For all experimental procedures, hONS cell lines were grown in 10% FBS in DMEM/F12 to provide sufficient numbers, harvested by enzymatic detachment, washed 3×in 10% FBS in DMEM/F12, and counted as described [7]. For metabolic functional assays, hONS cells were plated in 96 well plates at a density of 2500 cells per well in 100 µL of medium and allowed to attach overnight. For total protein and RNA extractions, hONS cultures were seeded into 6-well plates at a density of 1.2×10^5^ cells per well in 2 mL of medium. For all experiments, medium was changed the day following cell seeding, and replaced with either normal growth medium (basal PD compared to Control assessment), or replaced with medium containing DMSO as vehicle or indicated concentrations of NRF2 modulators.

L-SUL (Sigma) was dissolved in DMSO, and both t-BHQ (Sigma) and DEM (Sigma) were dissolved in ethanol. Final solvent concentration in tissue culture medium did not exceed 0.1%. Treated cells were compared to cells treated in parallel with equivalent volumes of vehicle in the medium.

### siRNA transfection

Control donor derived hONS cells were plated in antibiotic-free growth medium at densities indicated above and allowed to attach overnight. The following day, 7.5 µL of DharmaFECT reagent 1 transfection reagent (Dharmacon), or siRNAs (final concentration 50 nM) were diluted into 200 µL medium and incubated at room temperature for 5 minutes. Diluted siRNAs were added to diluted transfection reagent, and incubated for a further 20 minutes at room temperature, and 600 mL of normal growth medium was added to the mixture. Transfection mixture volumes were scaled as appropriate. Medium was aspirated from the cells, and prepared liposomes added to appropriate wells (1 mL per well for 6 well plates, 50 µL per well for 96 well plates) and the cells incubated at 37°C in 5% CO_2_. Five hrs post addition of liposomes, an equal volume of normal growth medium was added to each well, and the transfection continued overnight. The following day, the liposomes were removed, and replaced with normal growth medium, or with medium supplemented with vehicle or NRF2 inducing compounds as appropriate. NRF2 target sequences (Dharmacon) were UAAAGUGGCUGCUCAGAAU (NRF2 siRNA #09) and GAGUUACAGUGUCUUAAUA (NRF2 siRNA #10). As a negative control, a non-targeting sequence (Dharmacon) was used. All assays were conducted 48 hrs post-transfection.

### Immunoblot

Cells were plated and treated as described above. Following appropriate incubation times, total protein lysates were prepared using SDS-PAGE lysis buffer containing 1× HALT protease/phosphatase inhibitors (Pierce) and 10–15 µg total protein per lane assayed by SDS-PAGE and immunoblot as described [76]. Membranes were blocked in 5% milk in TBST for 1 hr at room temperature. Primary antibodies were incubated overnight at 4°C and included anti-NQO1 (1∶3000, Stressgen), anti-KEAP1 (1∶250, R&D Systems), and anti-NRF2 (1∶1000, Epitomics). Anti-GAPDH (1∶10000, Trevigen) and anti-β-tubulin (1∶2000, Sigma) were used as loading controls and were incubated at room temperature for 2 hrs. Antibody binding was detected and visualised using either anti-rabbit-IgG or anti-mouse-IgG HRP-conjugated secondary antibodies, ECL reagent (Millipore) and a digital imaging station (VersaDoc, BioRad).

### Nuclear extraction and DNA binding assay

Nuclear extracts were prepared from DMSO or L-SUL treated cultures using a commercially available kit following the manufacturer's instructions (Pierce) with the inclusion of an additional wash of the nuclear fraction in CERI buffer prior to nuclei lysis with NER buffer. Equal amounts of extract were electrophoresed on 4–20% Tris-glycine gradient SDS-PAGE gels (BIORAD), proteins transferred to PVDF and immunoblotted for NRF2 as described above. Histone H3 antibody (1∶1000, Cell Signalling Technology) was used as a loading control. NRF2 DNA binding was assessed using an ELISA-based assay using immobilised oligonucleotides corresponding to the consensus ARE motif from the human *NQO1* promoter according to the manufacturer's instructions (Active Motif). Briefly, equal amounts of nuclear extract were incubated with the oligonucleotide for 1 hr at RT. After washing, bound NRF2 was detected using the supplied NRF2 antibody followed by peroxidase-conjugated secondary antibody. Antibody binding was detected by addition of developing solution for 15 min at which time the reaction was stopped, and the absorbance at 450 nm recorded using 690 nm as a reference wavelength. Each cell line was assayed twice in triplicate, and data presented as fold-increase in DNA binding activity compared to respective DMSO treated control signal.

### Cell-based metabolic assays

Cells were plated and treated as described above. Following appropriate incubation times, metabolic functions were assessed essentially as per manufacturer supplied protocols, with only minor modifications as described previously [7]. For determination of total glutathione content, TCEP (1 mM final concentration) was added to the reaction buffer. Chymotrypsin-like, trypsin-like and caspase-like proteasome activities were all determined using the same protocols, with different activities based on substrate selectivity (Promega). For all assays, average background signal was subtracted from all other values for each assay, and data were normalised to DNA content (determined via CyQUANT-NF assay, Invitrogen). For each cell line and assay, triplicate values from duplicate experiments were averaged and a signal:DNA content ratio determined.

H_2_O_2_ levels in cell culture medium was assayed using 10-acetyl-3,7-dihydroxyphenoxazine (ADHP), a highly sensitive and stable probe for H_2_O_2_ which in the presence of horseradish peroxidase, reacts with H_2_O_2_ to produce resorufin. Briefly, 50 µL of prepared assay reagent (40 nM ADHP and 0.8 µg/mL peroxidase in PBS) was added to 100 µL of cell culture medium. After incubation at RT for 10 min in darkness, fluorescence emission at 590 nm was recorded after excitation at 530 nm using a microplate reader.

### Real-time PCR

RNA was extracted using commercially available kits (QIAGEN). Total RNA (2 µg) was reverse transcribed using SuperScript III first-strand synthesis system (Invitrogen). Levels of mRNAs were quantified and normalised against *EEF1A1* using a relative quantification approach with primer efficiency correction as described [7]. PCR conditions were 95°C for 10 minutes followed by 40 cycles at 95°C for 15 seconds, 58°C for 10 seconds and 72°C for 10 seconds. Primer sequences were *CYP1A1;* F-cacatgctgaccctgggaaag, R-ggtgtggagccaattcggatc; *CYP1B1*; F-cggccactatcactgacatc, R-ctcgagtctgcacatcagga; *NQO1*; F-cagctcaccgagagcctagt, R-gagtgagccagtacgatcagtg; *GCLC*; F-atgccatgggatttggaat, R-agatatactgcaggcttggaatg; *GCLM*; F-tgggcacaggtaaaaccaa, R-cagtcaaatctggtggcatc; *NRF2*; F-acacggtccacagctcatc, R-tgtcaatcaaatccatgtcctg; *EEF1A1*: F-ccccaggacacagagacttt, R-gcccattcttggagatacca.

### Microarray hybridisation and analysis

Whole genome arrays were performed on 16 Control and 13 PD hONS cultures, with each cell line treated with either DMSO or 2.5 µM L-SUL for 24 hr prior to RNA extraction (described above). RNA quantity and quality was accessed by Nanodrop (Thermo Scientific) and Agilent 2100 Bioanalyser (RNA Nano chips). Samples were labelled with Ambion Illumina RNA amplification kit using 500 ng of RNA and a 4 h IVT as per the manufactures instructions (Ambion). Labelled cRNA (750 ng) was hybridized to Illumina Human-HT-12 version 3 BeadChips (Illumina) for 16 hours and scanned on an Illumina Beadstation. Raw bead data were summarized in GenomeStudio V2009.1 and exported with no additional processing. Raw summarized data were background corrected and quantile normalized using the lumi package (http://www.bioconductor.org/packages/2.0/bioc/vignettes/lumi/inst/doc/lumi.pdf) in R/BioConductor. Normalised data were imported into and analysed in Genespring GX 7.3.1. software (Agilent Technologies). Data were filtered using Illumina detection p-value. A probe was included in further analysis if it had a detection p-value of ≤0.01 in 25% of samples within the four groups (PD vehicle, Control vehicle, PD L-SUL treated and Control L-SUL treated). Cell line expression patterns were investigated using principal component analysis. Differential expression was determined using ANOVA comparing Control and PD hONS transcriptomes from either vehicle or L-SUL treated cultures. Pathway over-representation analysis was determined using right-tailed Fisher's exact test using Ingenuity Pathway Analysis (IPA) 8.5 (Ingenuity Systems). Microarray data was deposited into ArrayExpress (Accession Number E-TABM-879), and is MIAME compliant.

## Supporting Information

Figure S1 **Cell metabolism deficiencies in PD-patient derived hONS cultures. A–F**. hONS cultures established from Control donors (N = 26) or from PD patients (N = 28) were assessed for levels of reduced glutathione (GSH) content, MTS metabolism, ATP content, chymotrypsin-like proteasome activity, caspase-3/7 activity and membrane integrity (LDH activity). Each circle (Cont) or square (PD) represents the average value for each assay after normalisation to DNA content for a given cell line, each assayed in triplicate on two occasions. Bars show mean and standard error of the mean (s.e.m.). Statistical significance was assessed using an unpaired two-tailed t-test with Welch's correction, with p<0.05 considered significant. Resulting p-values are shown above each graph. **G.** H_2_O_2_ levels in culture medium from either Control or Patient hONS cultures (N = 8 in each group) at 24, 48, 72 and 96 hours after cell seeding. Results are representative of 2 independent experiments that showed similar results. Statistical significance was assessed on time-point matched data between Controls and PD Patient values using an unpaired two-tailed t-test with Welch's correction, with p<0.05 considered significant; *, p<0.05; **, p<0.01.
(TIF)

Figure S2 **Lack of correlations of hONS cell metabolism with donor age or cell line passage number. A**. Gender of hONS cell line donors. B–C. Age (**B**) and cell line passage number (**C**) did not differ between Control and PD patient groups (p = 0.076 and p =  0.84 respectively, two tailed-t-test with p<0.05 considered significant). Each circle (control) or square (PD) represents an individual donor. Lack or correlation of cell metabolic function and donor age (**D–I**) or cell line passage number (**J–O**). Each circle represents data from an individual donor plotted against that donors' age or the number of passages for the derived cell line prior to assay. Linear regression analyses were conducted in Prizm 5 for Windows (GraphPad).
(TIF)

Figure S3 **Comparison of known NRF2 activators.** tBHQ (100 µM) and DEM (50 µM) were assessed for their ability to alter PD-associated metabolic deficiencies in Control (N = 3) and PD (N = 3) hONS cell lines. Cells were treated with indicated compounds for 24 hr prior to determination of reduced glutathione (GSH) content (**A–B**), total GSH content (**C–D**), or MTS metabolism (**E–F**). Bars show mean and s.e.m., with data presented as fold change compared to vehicle treated cultures for each cell line. Statistical significance was determined by ANOVA. *, p<0.05; **, p<0.01; ***, p<0.001; N.S.  =  not significant.
(TIF)

Figure S4 **Induction of GSH and MTS metabolism in Control donor-hONS cultures.** Control donor-derived hONS cultures (N = 17) were treated with 2.5 µM L-SUL for 24 hr prior to assessment of cell metabolic functions, including MTS metabolism (**A**), total and reduced glutathione (GSH) content (**B–C**), chymotrypsin-like, trypsin-like and caspase-likeproteasome activities (**D–F**), ATP content (**G**), caspase3/7 activity (**H**) and membrane integrity/LDH activity (**I**). Each circle (veh) or square (L-SUL treatment) represents data obtained from triplicate plates for an individual cell line. Error bars show mean and s.e.m.. Statistical significance was determined by two-tailed t-test with Welch's correction of vehicle versus L-SUL treated values, with p<0.05 considered significant. Resulting p-values are shown above each graph. **J.** Immunoblot analysis of total protein lysates from 2 Control and 3 PD hONS cultures treated with either DMSO (−) or 2.5 µM L-SUL (+) for 48 hrs. The protein being detected is shown to the left of each gel panel.
(TIF)

Figure S5 **Principle component analysis of mRNA expression changes in L-SUL treated hONS cultures.** Principle component analysis plot of the behaviour of each cell line sample relative to the major drivers of gene expression. The components are component 1: vehicle treatment, component 2: L-SUL treatment and component 3: disease status. This plot shows that L-SUL treatment is the primary driver of gene expression differences between groups, but there is also some segregation of the samples on disease status, though this is less well defined.
(TIF)

Figure S6 **Transcriptomic analysis identified discrete mRNA sets induced by L-SUL in Control versus PD hONS cultures. A**. Graphical representation of the `NRF2-mediated oxidative stress response' Ingenuity pathway annotation mRNA set post-L-SUL treatment, indicating those mRNAs either similarly expressed (clear icons), more highly expressed in PD hONS cultures (red icons) or more highly expressed in Control cultures (green icons). mRNAs identified as differentially expressed between Control and PD hONS cultures in vehicle treated cultures are indicated (red background shading), with those no longer differentially expressed in L-SUL cultures indicated (blue background shading). **B**. Graphical representation of the `PI3K / AKT signalling' Ingenuity pathway annotation mRNA set post-L-SUL treatment, indicating those mRNAs either similarly expressed (clear icons), more highly expressed in PD hONS cultures (red icons) or more highly expressed in Control cultures (green icons).
(TIF)

Table S1 **Individual PD-derived hONS cell line metabolic and qRT-PCR responses to 24** **hr treatment with 2.5** µ**M L-SUL.**
(DOC)

Table S2 **Gene expression changes in Control and PD-derived hONS cell lines.**
(XLS)

Table S3 **Fold-change gene expression changes in Vehicle-treated hONS cell lines.**
(XLS)

Table S4 **Fold-change gene expression changes in L-SUL-treated hONS cell lines.**
(XLS)

Table S5 **Geneset enrichment of Pathway Annotations based on Disease Status or Treatment Status.**
(XLS)
