# Supplementary figures and images for: NRF2 Activation Restores Disease Related Metabolic Deficiencies in Olfactory Neurosphere-Derived Cells from Patients with Sporadic Parkinson's Disease
Source: PLoS One. 2011 Jul 1;6(7):e21907. doi: 10.1371/journal.pone.0021907 (PMC3128624; doi:10.1371/journal.pone.0021907)

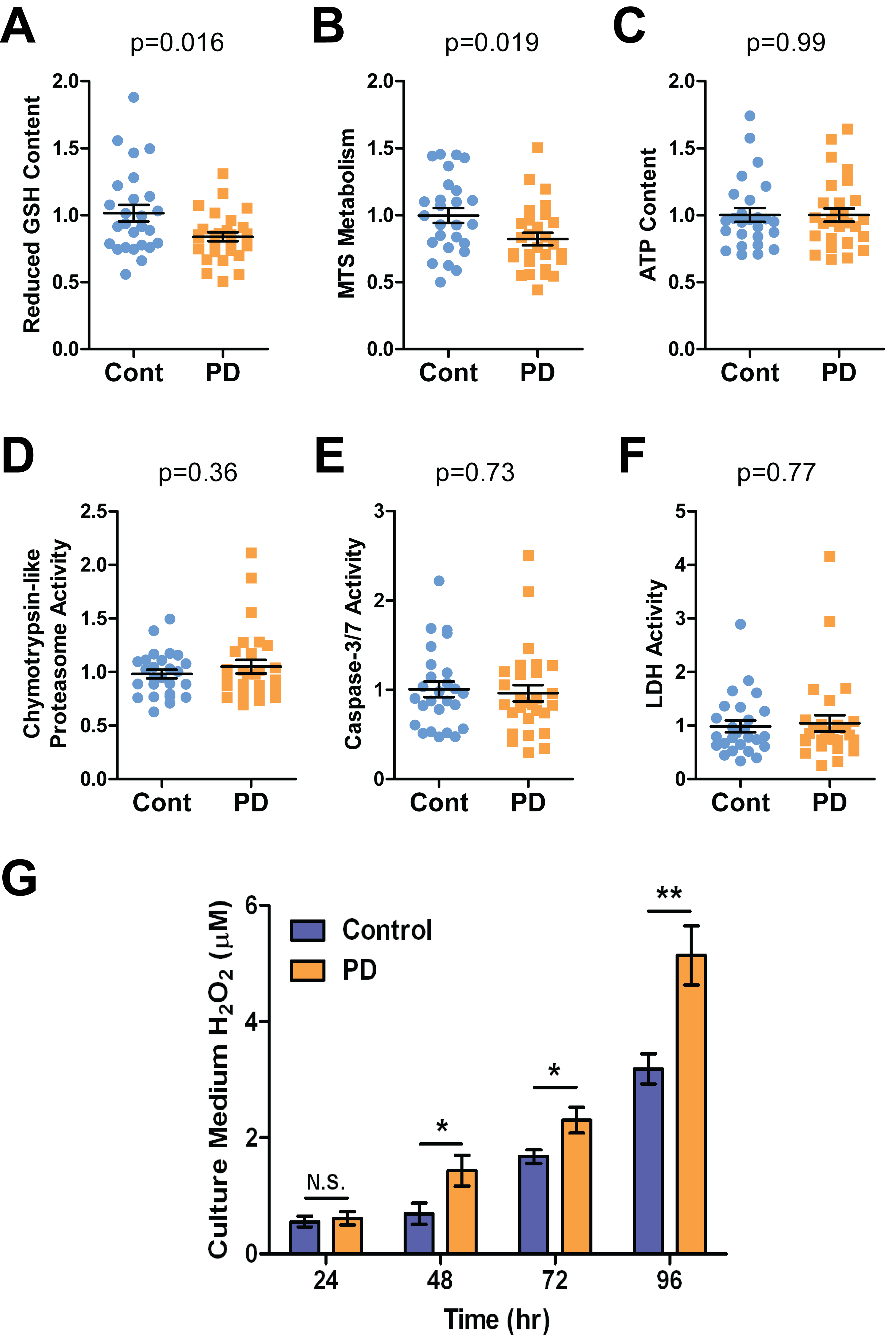

Supplement: Figure S1 — Cell metabolism deficiencies in PD-patient derived hONS cultures. A–F. hONS cultures established from Control donors (N = 26) or from PD patients (N = 28) were assessed for levels of reduced glutathione (GSH) content, MTS metabolism, ATP content, chymotrypsin-like proteasome activity, caspase-3/7 activity and membrane integrity (LDH activity). Each circle (Cont) or square (PD) represents the average value for each assay after normalisation to DNA content for a given cell line, each assayed in triplicate on two occasions. Bars show mean and standard error of the mean (s.e.m.). Statistical significance was assessed using an unpaired two-tailed t-test with Welch's correction, with p<0.05 considered significant. Resulting p-values are shown above each graph. G. H2O2 levels in culture medium from either Control or Patient hONS cultures (N = 8 in each group) at 24, 48, 72 and 96 hours after cell seeding. Results are representative of 2 independent experiments that showed similar results. Statistical significance was assessed on time-point matched data between Controls and PD Patient values using an unpaired two-tailed t-test with Welch's correction, with p<0.05 considered significant; *, p<0.05; **, p<0.01. (TIF) [file pone.0021907.s001.tif]

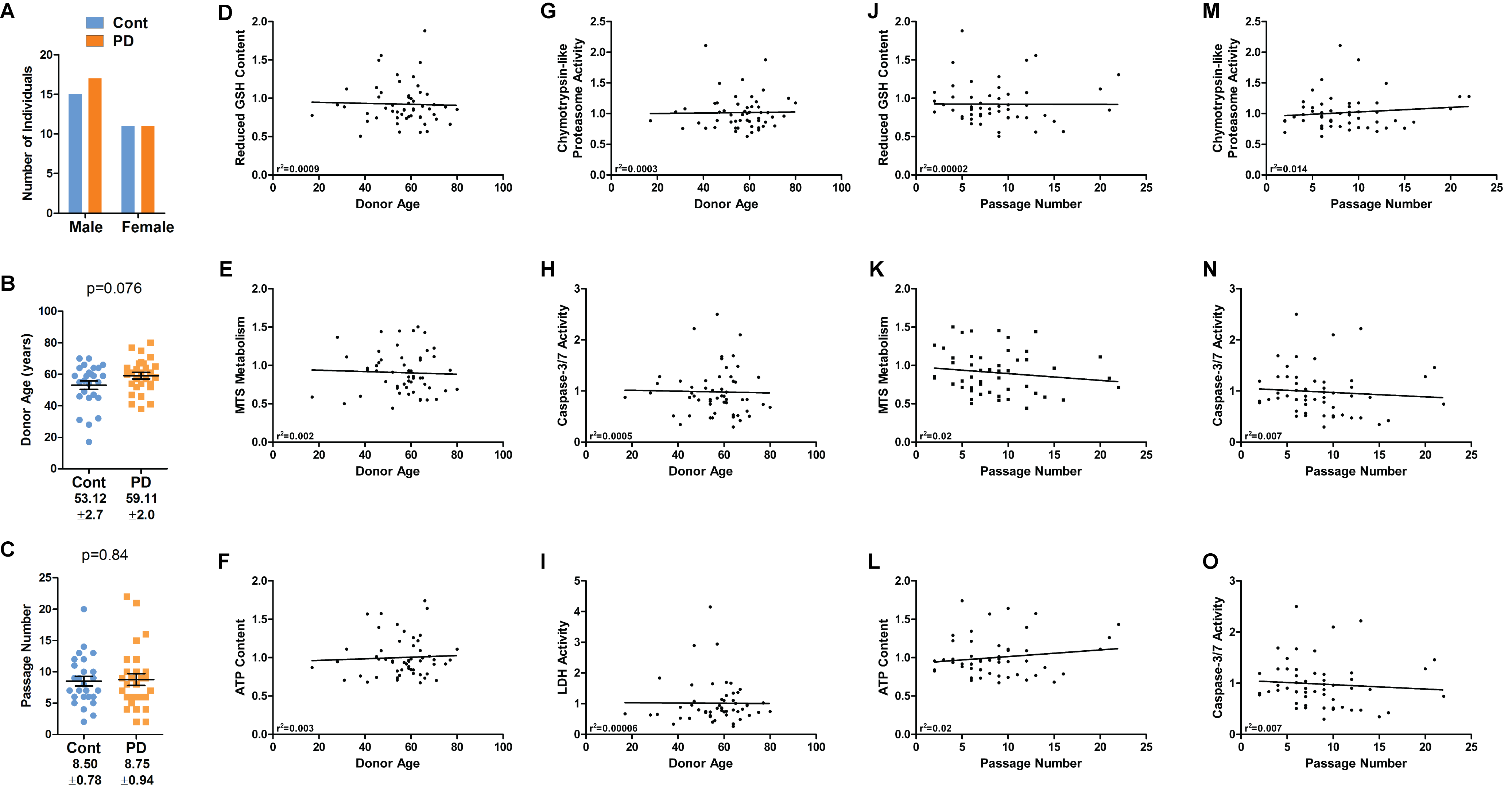

Supplement: Figure S2 — Lack of correlations of hONS cell metabolism with donor age or cell line passage number. A. Gender of hONS cell line donors. B–C. Age (B) and cell line passage number (C) did not differ between Control and PD patient groups (p = 0.076 and p = 0.84 respectively, two tailed-t-test with p<0.05 considered significant). Each circle (control) or square (PD) represents an individual donor. Lack or correlation of cell metabolic function and donor age (D–I) or cell line passage number (J–O). Each circle represents data from an individual donor plotted against that donors' age or the number of passages for the derived cell line prior to assay. Linear regression analyses were conducted in Prizm 5 for Windows (GraphPad). (TIF) [file pone.0021907.s002.tif]

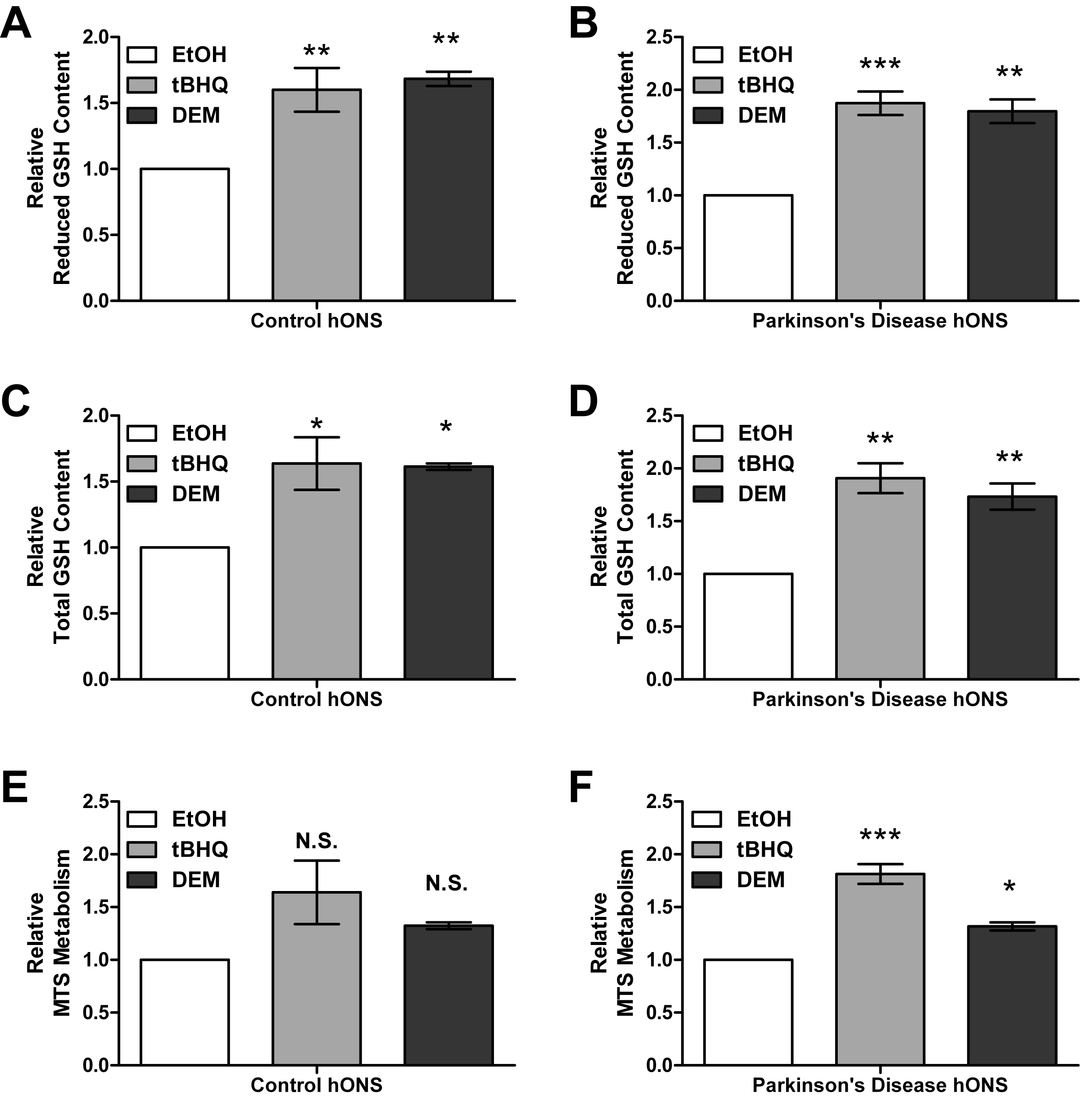

Supplement: Figure S3 — Comparison of known NRF2 activators. tBHQ (100 µM) and DEM (50 µM) were assessed for their ability to alter PD-associated metabolic deficiencies in Control (N = 3) and PD (N = 3) hONS cell lines. Cells were treated with indicated compounds for 24 hr prior to determination of reduced glutathione (GSH) content (A–B), total GSH content (C–D), or MTS metabolism (E–F). Bars show mean and s.e.m., with data presented as fold change compared to vehicle treated cultures for each cell line. Statistical significance was determined by ANOVA. *, p<0.05; **, p<0.01; ***, p<0.001; N.S. = not significant. (TIF) [file pone.0021907.s003.tif]

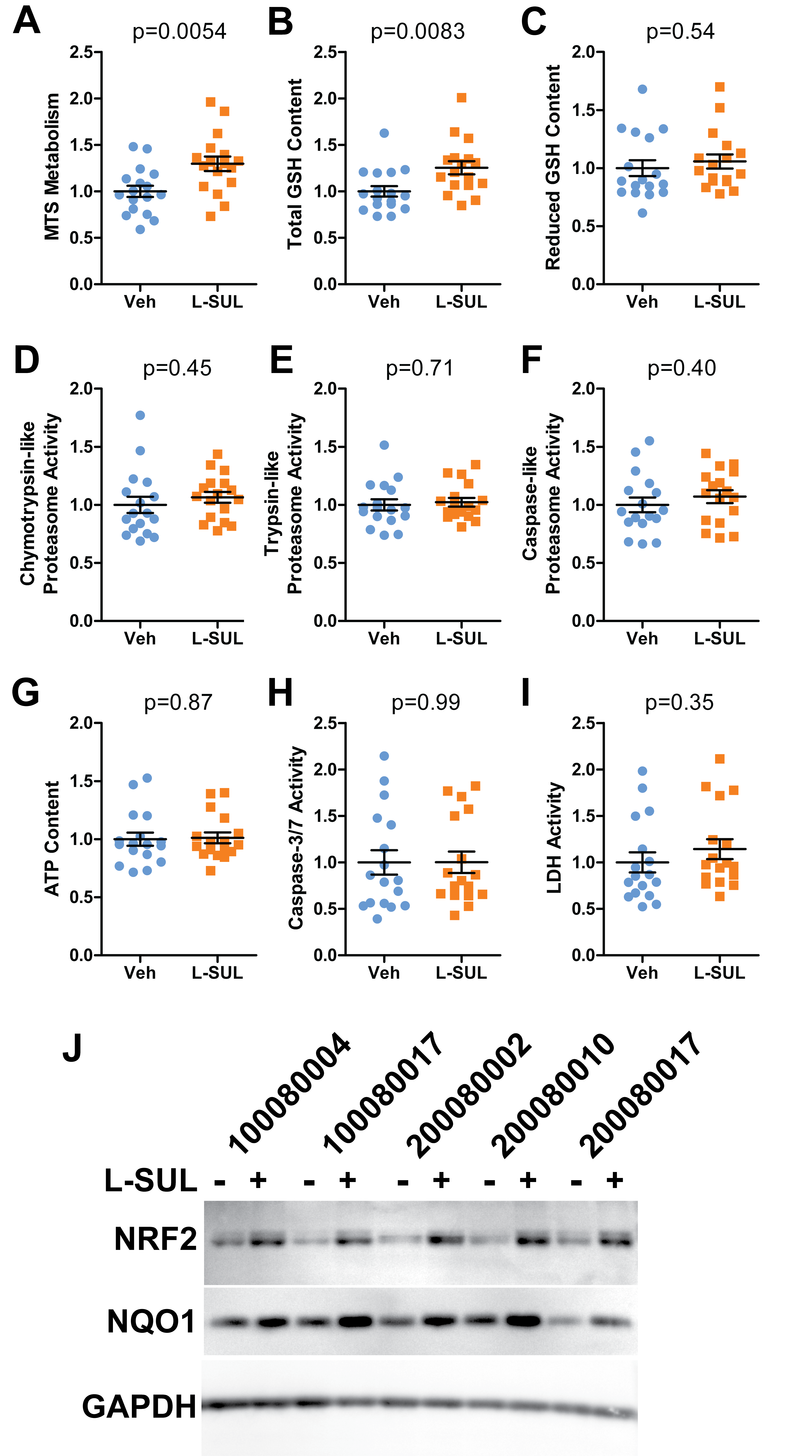

Supplement: Figure S4 — Induction of GSH and MTS metabolism in Control donor-hONS cultures. Control donor-derived hONS cultures (N = 17) were treated with 2.5 µM L-SUL for 24 hr prior to assessment of cell metabolic functions, including MTS metabolism (A), total and reduced glutathione (GSH) content (B–C), chymotrypsin-like, trypsin-like and caspase-likeproteasome activities (D–F), ATP content (G), caspase3/7 activity (H) and membrane integrity/LDH activity (I). Each circle (veh) or square (L-SUL treatment) represents data obtained from triplicate plates for an individual cell line. Error bars show mean and s.e.m.. Statistical significance was determined by two-tailed t-test with Welch's correction of vehicle versus L-SUL treated values, with p<0.05 considered significant. Resulting p-values are shown above each graph. J. Immunoblot analysis of total protein lysates from 2 Control and 3 PD hONS cultures treated with either DMSO (−) or 2.5 µM L-SUL (+) for 48 hrs. The protein being detected is shown to the left of each gel panel. (TIF) [file pone.0021907.s004.tif]

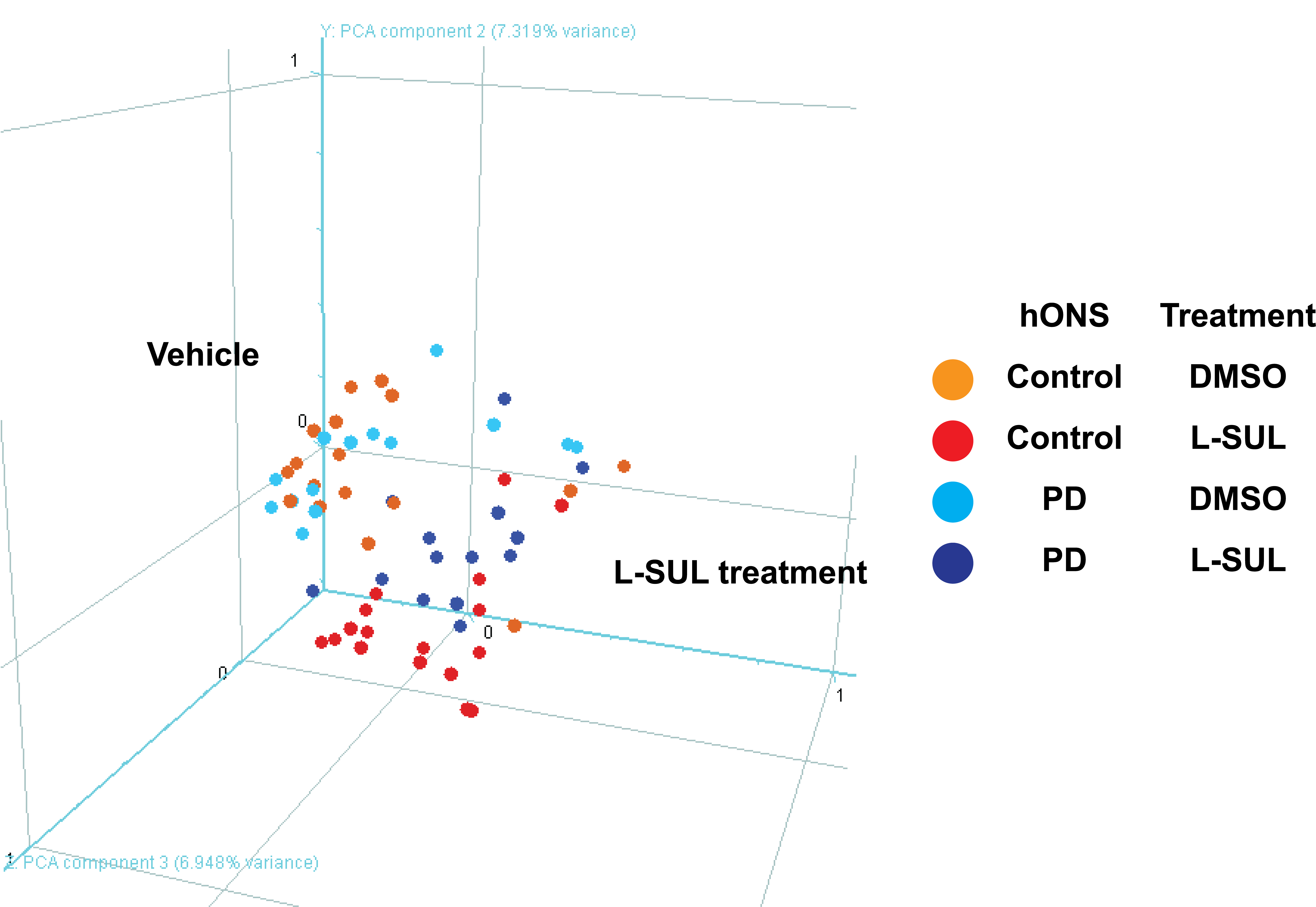

Supplement: Figure S5 — Principle component analysis of mRNA expression changes in L-SUL treated hONS cultures. Principle component analysis plot of the behaviour of each cell line sample relative to the major drivers of gene expression. The components are component 1: vehicle treatment, component 2: L-SUL treatment and component 3: disease status. This plot shows that L-SUL treatment is the primary driver of gene expression differences between groups, but there is also some segregation of the samples on disease status, though this is less well defined. (TIF) [file pone.0021907.s005.tif]

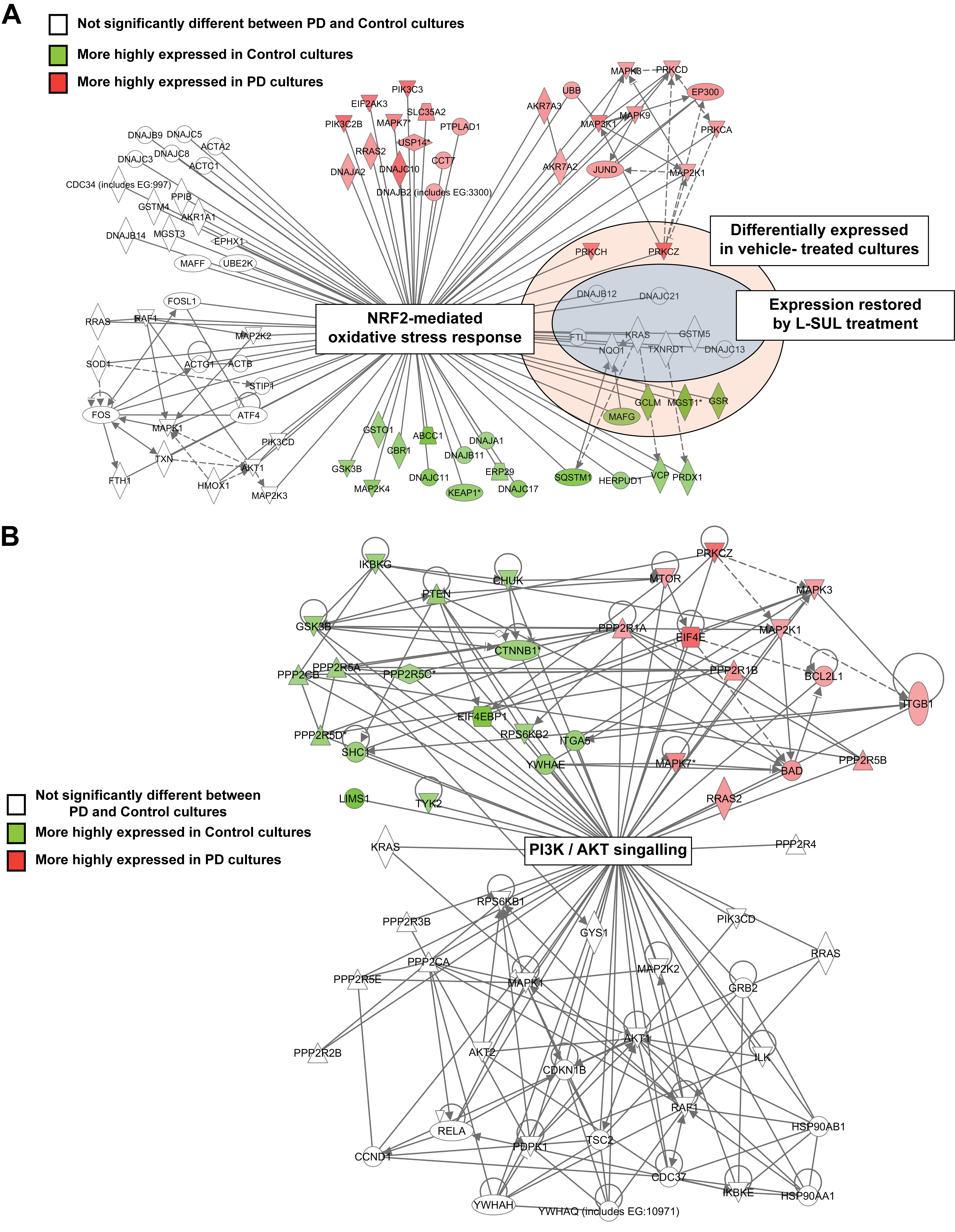

Supplement: Figure S6 — Transcriptomic analysis identified discrete mRNA sets induced by L-SUL in Control versus PD hONS cultures. A. Graphical representation of the `NRF2-mediated oxidative stress response' Ingenuity pathway annotation mRNA set post-L-SUL treatment, indicating those mRNAs either similarly expressed (clear icons), more highly expressed in PD hONS cultures (red icons) or more highly expressed in Control cultures (green icons). mRNAs identified as differentially expressed between Control and PD hONS cultures in vehicle treated cultures are indicated (red background shading), with those no longer differentially expressed in L-SUL cultures indicated (blue background shading). B. Graphical representation of the `PI3K / AKT signalling' Ingenuity pathway annotation mRNA set post-L-SUL treatment, indicating those mRNAs either similarly expressed (clear icons), more highly expressed in PD hONS cultures (red icons) or more highly expressed in Control cultures (green icons). (TIF) [file pone.0021907.s006.tif]
