# Supplementary material for: NRF2 Activation Restores Disease Related Metabolic Deficiencies in Olfactory Neurosphere-Derived Cells from Patients with Sporadic Parkinson's Disease
Source: PLoS One. 2011 Jul 1;6(7):e21907. doi: 10.1371/journal.pone.0021907 (PMC3128624; doi:10.1371/journal.pone.0021907)
Supplement: Table S1 — Individual PD-derived hONS cell line metabolic and qRT-PCR responses to 24 hr treatment with 2.5 µM L-SUL. (DOC) [file pone.0021907.s007.doc]

**Table S1. Individual PD-derived hONS cell line metabolic and qRT-PCR responses to 24 hr treatment with 2.5 μ**M L-SUL

|  | **Metabolic/Functional Assays** | | | | | | | | | | | | | | | | | | **qRT-PCR** | | | | | | | |
| --- | --- | --- | --- | --- | --- | --- | --- | --- | --- | --- | --- | --- | --- | --- | --- | --- | --- | --- | --- | --- | --- | --- | --- | --- | --- | --- |
| **Cell Line**  **Donor (Sex/Age)** | **MTS metabolism** | | **Total GSH** | | **Reduced GSH** | | **ATP content** | | **Chymotrypsin-like** | | **Trypsin-like** | | **Caspase-like** | | **LDH** | | **Caspase-3/7** | | **NRF2** | | **NQO1** | | **GCLC** | | **GCLM** | |
|  | Veh | L-SUL | Veh | L-SUL | Veh | L-SUL | Veh | L-SUL | Veh | L-SUL | Veh | L-SUL | Veh | L-SUL | Veh | L-SUL | Veh | L-SUL | Veh | L-SUL | Veh | L-SUL | Veh | L-SUL | Veh | L-SUL |
| 200060001  M/77 | 1.07 | 1.41 | 0.85 | 1.04 | 0.69 | 0.69 | 0.90 | 0.94 | 1.11 | 1.07 | 0.88 | 0.92 | 0.94 | 0.99 | 0.95 | 1.17 | 0.57 | 0.50 | 0.55 | 0.54 | 1.56 | 1.32 | 1.55 | 1.49 | 3.56 | 3.55 |
| 200060004  F/68 | 1.35 | 1.48 | 1.13 | 1.22 | 1.09 | 0.94 | 1.44 | 1.28 | 1.68 | 1.47 | 1.80 | 1.52 | 2.35 | 2.44 | 1.35 | 1.52 | 1.56 | 1.18 | 0.36 | 0.70 | 0.60 | 0.87 | 0.60 | 2.28 | 0.53 | 2.68 |
| 200060007  M/42 | 1.08 | 1.38 | 0.83 | 1.15 | 0.83 | 0.93 | 1.41 | 1.37 | 1.88 | 1.93 | 1.58 | 1.83 | 2.36 | 2.74 | 0.83 | 1.02 | 0.91 | 0.85 | 0.87 | 0.47 | 1.67 | 0.76 | 2.23 | 1.19 | 3.59 | 1.64 |
| 200070001  F/47 | 1.23 | 1.54 | 0.92 | 1.16 | 1.12 | 1.10 | 1.00 | 0.98 | 0.92 | 0.83 | 0.88 | 0.87 | 0.88 | 0.87 | 1.00 | 1.21 | 0.69 | 0.69 | 0.53 | 0.47 | 1.47 | 3.95 | 2.25 | 3.26 | 3.11 | 6.98 |
| 200070002  M/80 | 0.79 | 1.08 | 0.89 | 1.20 | 0.89 | 0.93 | 1.02 | 1.05 | 1.05 | 1.17 | 0.94 | 1.04 | 1.11 | 1.18 | 0.69 | 0.78 | 0.52 | 0.47 | 0.67 | 0.78 | 1.79 | 6.09 | 1.79 | 2.83 | 4.46 | 11.27 |
| 200070003  F/53 | 0.50 | 0.60 | 0.92 | 1.23 | 0.94 | 1.02 | 0.86 | 0.96 | 0.77 | 0.77 | 0.85 | 0.92 | 0.59 | 0.85 | 0.68 | 0.94 | 0.89 | 0.97 | 0.67 | 0.63 | 1.39 | 5.08 | 1.41 | 1.90 | 4.21 | 7.97 |
| 200080001  F/58 | 0.80 | 1.09 | 1.05 | 1.25 | 0.96 | 0.88 | 0.90 | 0.96 | 0.64 | 0.73 | 0.83 | 0.94 | 0.82 | 0.83 | 1.69 | 1.94 | 1.32 | 1.26 | 0.81 | 0.86 | 1.49 | 6.10 | 2.23 | 3.32 | 4.97 | 12.35 |
| 200080003  F/53 | 0.93 | 1.22 | 0.95 | 1.16 | 0.87 | 0.81 | 0.93 | 0.89 | 0.87 | 0.89 | 0.78 | 0.80 | 0.66 | 0.69 | 0.80 | 0.87 | 0.84 | 0.69 | 0.61 | 0.73 | 1.24 | 5.99 | 1.45 | 2.42 | 3.78 | 10.06 |
| 200080005^*^  M/69 | 0.91 | 1.23 | 1.11 | 1.98 | 1.04 | 1.29 | 1.00 | 1.07 | 0.96 | 1.23 | 1.03 | 1.15 | 0.88 | 0.95 | 0.97 | 1.21 | 0.99 | 0.94 | 0.50 | 0.54 | 1.60 | 6.19 | 1.86 | 3.12 | 4.03 | 10.11 |
| 200080007  M/57 |  |  |  |  |  |  |  |  |  |  |  |  |  |  |  |  |  |  | 0.36 | 0.37 | 1.26 | 4.09 | 1.60 | 2.58 | 2.68 | 6.58 |
| 200080010  F/60 | 1.06 | 1.50 | 1.03 | 1.37 | 0.96 | 1.06 | 0.74 | 0.75 | 0.51 | 0.53 | 0.74 | 0.84 | 0.45 | 0.51 | 0.91 | 1.19 | 1.26 | 1.00 | 0.46 | 0.44 | 1.24 | 4.35 | 1.40 | 2.36 | 4.73 | 11.30 |
| 200080013  F/58 | 0.96 | 0.96 | 1.23 | 1.26 | 1.39 | 1.17 | 1.11 | 0.76 | 1.24 | 0.76 | 0.88 | 0.87 | 0.67 | 0.52 | 1.00 | 0.96 | 1.38 | 0.85 | 0.38 | 0.35 | 1.00 | 3.24 | 1.40 | 2.06 | 3.24 | 7.43 |
| 200080016  M/47 | 1.22 | 1.37 | 1.14 | 1.54 | 1.40 | 1.38 | 1.04 | 0.99 | 0.77 | 0.75 | 1.01 | 0.94 | 0.89 | 0.75 | 1.38 | 1.35 | 1.11 | 0.86 | 0.39 | 0.47 | 1.68 | 5.50 | 2.59 | 3.87 | 4.33 | 10.54 |
| 200080022  M/54 | 1.13 | 1.55 | 0.92 | 1.35 | 0.85 | 0.92 | 0.73 | 0.70 | 0.69 | 0.72 | 0.92 | 0.82 | 0.58 | 0.68 | 0.79 | 0.95 | 1.05 | 0.83 |  |  |  |  |  |  |  |  |
| 200090003  F/65 | 0.96 | 1.17 | 1.03 | 1.37 | 0.94 | 0.95 | 0.92 | 0.91 | 0.91 | 0.79 | 0.87 | 0.89 | 0.81 | 0.77 | 0.95 | 1.16 | 0.90 | 0.72 |  |  |  |  |  |  |  |  |
| **Average** | **1.00** | **1.26** | **1.00** | **1.31** | **1.00** | **1.01** | **1.00** | **0.97** | **1.00** | **0.97** | **1.00** | **1.02** | **1.00** | **1.05** | **1.00** | **1.16** | **1.00** | **0.84** | **0.55** | **0.57** | **1.38** | **4.12** | **1.72** | **2.51** | **3.63** | **7.88** |

^*^Average of duplicate cell lines established from the same donor (cell lines 200080005, 200080006)
